# Assessment of the correlation between the nutrient load from migratory bird excrement and water quality by principal component analysis in a freshwater habitat

**DOI:** 10.1007/s11356-023-27065-3

**Published:** 2023-04-25

**Authors:** Piroska Tóth, Bálint Levente Tarcsay, Zsófia Kovács, Dan Traian Ionescu, Sándor Németh, Endre Domokos

**Affiliations:** 1 Sustainability Solutions Research Lab, Research Centre for Biochemical, Environmental and Chemical Engineering, University of Pannonia, Veszprém, Hungary; 2 Department of Process Engineering, University of Pannonia, Veszprém, Hungary; 3 National Laboratory for Water Science and Water Security, Research for Biochemical, Environmental and Chemical Engineering, Sustainability Solutions Research Lab, University of Pannonia, Veszprém, Hungary; 4 Department of Forest Engineering, Transilvania University of Brașov, Brașov, Romania

**Keywords:** Water quality assessment, Waterbird species, Principal component regression (PCR), Nutrient load, Water quality index (WQI), River, Bird migration

## Abstract

Waterbirds depend on a dispersed network of wetlands for their annual life cycle during migration. Climate and land use changes raise new concerns about the sustainability of these habitat networks, as water scarcity triggers ecological and socioeconomic impacts threatening wetland availability and quality. During the migration period, birds can be present in large enough numbers to influence water quality themselves linking them and water management in efforts to conserve habitats for endangered populations. Despite this, the guidelines within laws do not properly account for the annual change of water quality due to natural factors such as the migration periods of birds. Principal component analysis and principal component regression was used to analyze the correlations between the presence of a multitude of migratory waterbird communities and water quality metrics based on a dataset collected over four years in the Dumbrăvița section of the Homoród stream in Transylvania. The results reveal a correlation between the presence and numbers of various bird species and the seasonal changes in water quality. Piscivorous birds tended to increase the phosphorus load, herbivorous waterbirds the nitrogen load, while benthivorous duck species influenced a variety of parameters. The established PCR water quality prediction model showed accurate prediction capabilities for the water quality index of the observed region. For the tested data set, the method provided an *R*^2^ value of 0.81 and a mean squared prediction error of 0.17.

## Introduction

Clean water is vital to our health, communities, and economy. Clean water is life, health, food, leisure, and energy (Bruyninckx 2018). The urban sprawl, increase in population, and economic activity, however, lead to increased water demand and decreased water quality (Heidari et al. 2021).

The need for proper water quality results in strict regulations for the supervision of the chemical parameters of water, especially with regard to nitrogen and phosphorous compounds. In spite of this, natural sources of water pollution are unaccounted for in these regulations. The aim of this study is to investigate the impact of natural phenomena, such as bird migration, on water quality and chemical parameters in order to help set adaptive thresholds for water quality regulation.

The continuous improvement in quality of life is directly linked to the quality of the environment and especially the quality of water. Sustainable Development Goals (SDGs) should be taken into account to improve the condition of the environment by taking advantage of innovative technologies and the “data revolution” (Sebestyén et al. 2020; Mondejar et al. 2021). Around 40% of surface waters (rivers, lakes, and transitional and coastal waters) are in good ecological status or potential, and only 38% are in good chemical status. In the European Union, the significant pollution pressure types on surface water bodies are hydromorphological pressures (affecting 40% of water bodies), diffuse sources (38%), particularly from agriculture, and atmospheric deposition (38%), followed by point sources (18%) and water abstraction (6%) and over 1/3 of river basin districts are cross-border (European Environment Agency 2018).

Surface water quality is mainly influenced by various natural factors (precipitation, seasons, weather, catchment area, soil erosion, and biota) and anthropogenic factors (intensive agricultural activities and pesticide use, livestock production, domestic and industrial wastewater release, urban development, population growth, deforestation, illegal dumping, plastic pollution, etc.) (Carpenter et al. 1998; Brain and Anderson 2020). Rivers are among the world’s most vulnerable ecosystems (Vörösmarty et al. 2010).

Considerations for sustainable water management practices and proper water quality regulations are laid down in the Water Framework Directive (WFD). The fundamental objective of the WFD is to achieve the good ecological status for surface water and groundwater using a transboundary river basin approach, leaving it to authorities to set ecological objectives adapted to both water system characteristics and the driving forces that influence water quality (Wuijts et al. 2023). A cross-sectoral approach is used to try to reach the goals of the WFD, point sources, such as wastewater treatment plants got regulated, policies have been drafted to deal with diffuse sources such as agriculture; however, some natural factors, such as bird migration or the winter slowdown of microbial activity, are typically not taken into consideration. Nitrogen and phosphorus play a dominant role in the degradation of water quality in lakes, streams, and wetlands and are thus factors of key importance when classifying water quality according to the framework (Jolánkai et al. 2020; Yang et al. 2022).

The study of waterbirds (waterfowl, shorebirds, and wading birds) and water management have long been linked in efforts to conserve the habitat of endangered populations. Waterfowl are most at risk from climate and land-use change, habitat loss (Szép et al. 2012; Simkin et al. 2022), illegal hunting (Si et al. 2021), collisions, and electrocution by power lines and disease (Donnelly et al. 2022).

During the bird migration season, water bodies that serve as feeding and shelter sites for migratory waterbird communities (stopover sites) have particularly high importance. High-quality habitats support the long-term population stability of waterbirds (Ionescu et al. 2008). Birds, however, consume large amounts of food to maintain their body temperature, resulting in large amounts of excrement.

Bird excrements are a source of nutrient (particularly nitrogen and phosphorus compounds) and bacterial loads of water bodies (Marion et al. 1994; Hahn et al. 2007). Bird migration is a cyclical, natural phenomenon that increases nutrient loads, sedimentation rates, and coliform bacteria concentrations in water bodies, depending on the number and species of birds in the area, and must be taken into account in sustainable water management practices as described in the Water Framework Directive (WFD) (Wu et al. 2021).

There have been numerous studies on the role of waterbirds on nutrient loads in aquatic environments of varying importance and diversity throughout the world. Additionally, there have been studies recently which provide a comprehensive overview of ecologically derived nutrient thresholds in rivers that could be useful when setting nutrient targets. Technical guidance has been developed to enable countries to establish or review thresholds for phosphorus and nitrogen to support good ecological status (Poikane et al. 2021). However, few studies have been conducted on continental aquatic systems (Gere and Andrikovics 1992). Additionally, in most of these studies, estimates of waterbird pressures on surface waters are mostly limited to a single bird species in lakes and reservoirs (Somura et al. 2015).

In this study, we utilized principal component analysis (PCA) to observe the annual changes within chemical parameters of water in the Dumbrăvița section of the Homorod watercourse in the central part of the Brașov basin, which is part of the Olt river catchment area. Since 2006, the Dumbrăvița section of the Homorod watercourse is a Ramsar site, the only such site in Transylvania Province (the term Transylvania refers, from a political-geographical point of view, to the intra-Carpathian region, bounded by the Eastern Carpathians, the Southern Carpathians, and, to the west, by the Apuseni Mountains); since 2007, it is designated as a wetland area protected by the Natura 2000 (special protection area, SPA) as the Natura 2000 site “Dumbrăviţa—Rotbav—Măgura Codlei”—ROSPA0037. The Dumbrăvița fishing complex covers an area of around 450 hectares and is a system of reservoirs and fish ponds, surrounded by reeds, grassland, and willow groves. Today, this area provides a habitat for more than 100 species of birds during their annual migrations (Ionescu et al. 2019). The main human activities in the whole wetland area are aquaculture and fishing. The main species cultivated are Asian, such as, such as *Hypophthalmichthys nobilis*, *Hypophthalmichthys molitrix*, and *Ctenopharyngodon idella* but also the Eurasian species *Cyprinus carpio*. In addition, there are other small fish, some invasive, such as *Pseudorasbora parva*, food for many piscivorous birds (Ionescu et al. 2020).

## Materials and methods

During the investigation, the PCA multivariate statistical method was utilized for analyzing the annual changes within the chemical parameters of water as well as to establish a correlation between the presence of migratory bird species and water quality. As a way of providing a means to predict natural changes in water quality over the year, PCR was performed to establish a link between the presence of bird species and observed water quality index (WQI) values. An overview of the various metrics used for quantifying water quality as well as the basis of the employed multivariate statistical methods is provided in the following section. During the investigation, the water parameters required by the WFD (WFD 2000/60/EC) were observed. Water sampling was carried out according to the ISO 5667–1 standard by the Olt Water Basin Administration. The water quality measurements were performed by the national authorities according to international standards by the Olt Water Basin Administration (Administrația Bazinală de Apă Olt) during the period 2010–2019, including water temperature, pH, nitrate (NO_3_), nitrate-nitrogen (N-NO_3_), ammonium (NH_4_), ammonium-nitrogen (N-NH_4_), nitrite (NO_2_), nitrite-nitrogen (N-NO_2_), orthophosphate-phosphorus (P-PO_4_), total phosphorus (P), total nitrogen (N), total suspended solids, water flow, conductivity, dissolved oxygen, five-day carbonaceous biochemical oxygen demand (CBO_5_), chemical oxygen demand (CCO_Cr_), total suspended solids (TSS), and turbidity (Table 1). The fixed residue is the residue left after evaporating the water sample at 105 °C, organic and inorganic contaminants together. Chemical oxygen demand (CCO_Cr_) is the amount of oxygen taken from the oxidant during the chemical oxidation of organic matter and some inorganic compounds (i.e., nitrites, nitrates, and sulfates) using an excess amount of potassium dichromate, while the carbonaceous biochemical oxygen demand (CBO_5_) is the oxygen required by microorganisms for breaking down organic matter under standard laboratory procedures in five days at 20 °C, minus the nitrogenous oxygen demand fraction.

**Table 1:** Investigated variables for the PCA evaluation of annual changes within water quality

Variable	Unit	Variable	Unit
Carbonaceous oxygen demand (CBO_5_)	mg l^−1^	Total nitrogen concentration (N)	mg l^−1^
Chemical oxygen demand (CCO_Cr_)	mg l^−1^	Total phosphorous concentration (P)	mg l^−1^
Fixed residue (FR)	mg l^−1^	pH	-
Ammonium nitrogen concentration (N-NH_4_)	mg l^−1^	Orthophosphate phosphorous concentration (P-PO_4_)	mg l^−1^
Nitrite nitrogen concentration (N-NO_2_)	mg l^−1^	Total suspended solids (TSS)	mg l^−1^
Nitrate nitrogen concentration (N-NO_3_)	mg l^−1^		

Bird counts were carried out regularly at selected points. Fieldwork was carried out over the year, with intense times of data acquisition being the spring (15 February–15 May) and autumn (15 August–30 November) migration periods. The observation was carried out in the location defined by coordinates N 45° 43′ 1″, E 25° 24′ 38″.

For bird count and identification only standardized, non-invasive methods were used, choosing observation points from where you can best observe the entire colony (Domşa et al. 2014). Colonies and flocks were identified from the shore by vehicle and aerial drone surveys without significantly disturbing the birds. Waterbird populations were estimated using other methods (point counting and transects) for all waterbirds using the 2 × 2 square method. We used binoculars (10 × 48), scope (× 20–60), a professional camera, and a drone. The observation period was 2010–2020, but only the period 2016–2020 was included in the analysis. Based on the previous observations, the relative impact of certain bird species on the lake’s water quality was investigated. The correlation between the presence of migratory birds and the chemical parameters of water of the watercourse was observed during a 4-year period between 2016 and 2020. During the investigation, a databank was created that contained observations of the presence of more than 100 migratory bird species which could be found near the reservoirs. Among these 12 species were selected based on the severity of their environmental impact with regards to the concentration of phosphorus and nitrogen compounds within their excrement as well as their number during migration seasons (Manny et al. 1994; Marion et al. 1994; Morkune et al. 2020; Boros et al. 2021).

PCA was utilized to observe the annual change of natural pressure sources on surface water quality and later principal component regression (PCR) was utilized to establish a link between the presence of the twelve most dominant migratory bird species and water quality metrics.

### Water quality metrics

The Romanian national guidelines are described by the Romanian Ministry of Research and Innovation in Order 161/2006 (Ord. 161/2006 2006), and these were used as a basis for further evaluation. In order to give a comprehensive picture of water quality by a single value, a variety of indices have been devised. Among these, the CCME-WQI was selected for official use by the national authorities. The CCME-WQI with time series analysis was used to explore changes in water quality of the Dumbrăvița Reservoir and the Homoród stream.

The CCME-WQI tests the concentration of individual variables against the guideline values according to three different metrics, as shown in the Eq. 1.

1 $$\mathrm{CCME}-\mathrm{WQI}=100-\left(\frac{\sqrt{{F}_{1}^{2}+{F}_{2}^{2}+{F}_{3}^{2}}}{1.732}\right)$$

*F*1 is the ratio of variables that do not comply with the national guideline (“failed variables”) at least once relative to the total number of measured variables. *F*2 is the percentage of individual tests that do not comply with the guideline (“failed tests”) relative to the total number of tests. Finally, *F*3 considers the degree to of the failed test values differs from the guidelines. The value of the CCME-WQI index gives a numerical value to the state of water quality between 0 and 100 and is typically ranked into five categories: excellent: 95–100; good: 80–94; fair: 65–79; marginal: 45–64; poor: 0–44.

### Principal component analysis and principal component regression

PCA is a data dimensionality reduction and outlier identification technique (Gorban et al. 2008).

To explain the logic and derivation of PCA, the following notations will be utilized. Let ${\varvec{X}}\in {R}^{n\times p}$ be a matrix containing data standardized to zero mean and unit variance in each *i* = 1,..,*p* column where *n* is the number of observations and *p* is the number of observed variables. During the course of PCA, instead of analyzing the data with regards to the *p* variables, a transformation is applied to the data such that it can be viewed as a function of *m* variables, where ideally $m\ll p$ holds true (Jolliffe 2005; Jolliffe 2010).

This applied transformation is linear and determines a set of orthogonal vectors called loading vectors that are ordered by the amount of variance explained in the loading vector directions. To obtain the transformation of the sample covariance matrix ($\mathbf{V}\in {R}^{p\times p}$) of ***X*** is calculated according to Eq. 2.

2 $${\varvec{V}}=\frac{1}{n-1}{{\varvec{X}}}^{{\varvec{T}}}{\varvec{X}}$$

After performing singular value decomposition on the covariance matrix, Eq. 3 is acquired where ${\varvec{P}}\in {R}^{p\times p}$ is a matrix containing the so-called loading vectors as its columns which are the eigenvectors of **V** while ${\varvec{\Lambda}}\in {R}^{p\times p}$ is a matrix containing the eigenvalues of ${\varvec{V}}$ in its diagonal.

3 $${\varvec{V}}={\varvec{P}}\boldsymbol{\Lambda }{{\varvec{P}}}^{{\varvec{T}}}$$

The eigenvalues in matrix ${\varvec{\Lambda}}$ are sorted in descending order together with the corresponding eigenvectors in ${\varvec{P}}$. After this, the number of PCs to be retained (*m*) can be chosen based on various criteria, including the elbow rule or Kaiser’s condition (De Ketelaere et al. 2015).

After choosing an appropriate number of variables to be retained, the ${\varvec{P}}{\varvec{C}}\in {R}^{n\times m}$ values of the dataset can be calculated using the reduced matrix of loading vectors (${{\varvec{P}}}_{{\varvec{\varepsilon}}}\in {R}^{p\times m}$) containing the first *m* columns of ***P*** according to Eq. 4.

4 $${\varvec{P}}{\varvec{C}}={\varvec{X}}{{\varvec{P}}}_{{\varvec{\varepsilon}}}$$

After obtaining the ${{\varvec{P}}}_{{\varvec{\varepsilon}}}$ transformation matrix correlation between the original variables of the data set may be observed based on the loading values stored in the columns of ${{\varvec{P}}}_{{\varvec{\varepsilon}}}$. Similar loading scores for different variables indicate the correlation between said variables. Outliers of the data set can be analyzed using measures such as the *Q*-statistic or Hotelling’s *T*^2^ statistic. This can be done by evaluating the ${T}^{2}$ value of individual data points ($x$) according to Eq. 5. provided that $\boldsymbol{\Lambda }$ is invertible.

5 $${{\varvec{T}}}^{2}={{\varvec{x}}}^{{\varvec{T}}}{\varvec{P}}{\boldsymbol{\Lambda }}^{-1}{{\varvec{P}}}^{{\varvec{T}}}{\varvec{x}}$$

This value is then compared to the hypothesis threshold of the *T*^2^ statistic corresponding to a defined confidence level (α) as calculated per Eq. 6, in which $F\left(\alpha ,n,m\right)$ represents the *F*-distribution with parameters $n$ and $m$.

6 $${{\varvec{T}}}_{\boldsymbol{\alpha }}^{2}=\frac{{\varvec{m}}\left({\varvec{n}}-1\right)}{{\varvec{n}}-{\varvec{m}}}{\varvec{F}}\left(\boldsymbol{\alpha },{\varvec{n}},{\varvec{m}}\right)$$

Outliers to a given confidence level can be detected by checking if the *T*^2^ value of the data point exceeds the calculated ${T}_{\alpha }^{2}$ value.

Utilizing the established PC-s, a regression may be performed, which is referred to as principal component regression (PCR). PCR utilizes the ordinary least squares regression model using the principal components to establish a connection between the original data matrix ${\varvec{X}}\in {R}^{n\times p}$ and an arbitrary observed variable $y\in {R}^{n\times 1}$. The form of the regression model is shown in Eq. 7, where ${\varvec{\beta}}\in {R}^{p\times 1}$ is the parametric vector of the linear regression.

7 $$y={\varvec{X}}\upbeta$$

In the case of PCR, the regression is performed on the PCs which helps eliminate collinearity between the original variables and prevent numerical issues. Using the solution to the ordinary least squares regression problem, the parametric vector can be calculated according to Eq. 8 (Jolliffe I. 2005).

8 $${\upbeta ={\varvec{P}}}_{{\varvec{\varepsilon}}}{\left({\varvec{P}}{\varvec{C}}\boldsymbol{^{\prime}}{\varvec{P}}{\varvec{C}}\right)}^{-1}{\varvec{P}}{\varvec{C}}\boldsymbol{^{\prime}}y$$

To perform predictions utilizing the PCR method, generally, the observed data set is partitioned into multiple segments, one being used for training the other for validating the predictive capabilities of the estimator. To quantify the performance of the regression, the coefficient of determination (*R*^2^) and the mean squared prediction error (MSPE) metrics are generally used (Jolliffe 2010). The *R*^2^ provides a measure for the ratio of variation in the observed variable versus the variation which can be observed by utilizing the predictor variables. The MSPE metric, on the other hand, characterizes the absolute value of the mean squared and summed prediction error of the regression for the observed variable.

The two metrics can be calculated according to Eq. 9 and Eq. 10, respectively. In Eq. 8, $\widetilde{y}\in {R}^{n\times 1}$ represents predicted values of the observed variable calculated by using Eq. 7 and $\overline{y }$ represents the mean value of the observed data set $y$.

9 $${R}^{2}=1-\frac{\sum_{i}{\left({y}_{i}-{\widetilde{y}}_{i}\right)}^{2}}{\sum_{i}{\left({y}_{i}-\overline{y }\right)}^{2}}$$

In the case of Eq. 10, the symbol *E* denotes the expectance of the squared sum of residuals for the prediction.

10 $$\mathrm{MSPE}=E\left(\sum_{i}{\left({y}_{i}-{\widetilde{y}}_{i}\right)}^{2}\right)$$

Evaluation of a data set collected on the lake near Dumbrăvița, Romania, through PCA has been conducted, and conclusions have been drawn based on the observed dataset. The dataset contained samples which show the changes within the chemical parameters of water of the lake for 11 variables (total suspended solids, nitrite, nitrate concentration, pH, fixed residue, total nitrogen and nitrate compound concentration, biological and chemical oxygen demand, total phosphorous, and phosphate compound concentration) over the months for 4 years between 2016 and 2020. The list of the observed variables and their units is displayed in Table 1.

## Results

The tendencies of annual changes within the chemical parameters of water are analyzed. Correlations between the parameters pertaining to water quality are observed, and conclusions are drawn with regard to the impact of migration periods on water quality. Subsequently, the correlation between the presence of migratory birds and water quality is analyzed, and a model is established to predict the WQI of the water as a function of the number and species of migratory birds present over a time period.

### Evaluation of annual changes within water quality

In this subsection correlation between nutrient loads of birds and migration seasons was investigated, especially for the 12 dominant bird species of the region. According to the migration seasons, there were two peaks in nutrient loads of birds, one in early spring (March) and the other in late autumn (November). First, there was no direct relationship between nutrient loads from waterbirds and water quality parameters at small time scales, e.g., daily periods, but a direct relationship could be identified at monthly scales.

The monthly sum of nutrient loads by waterbirds was correlated with the monthly average concentration of investigated nitrogen and phosphorus nutrients.

**Fig. 1:**
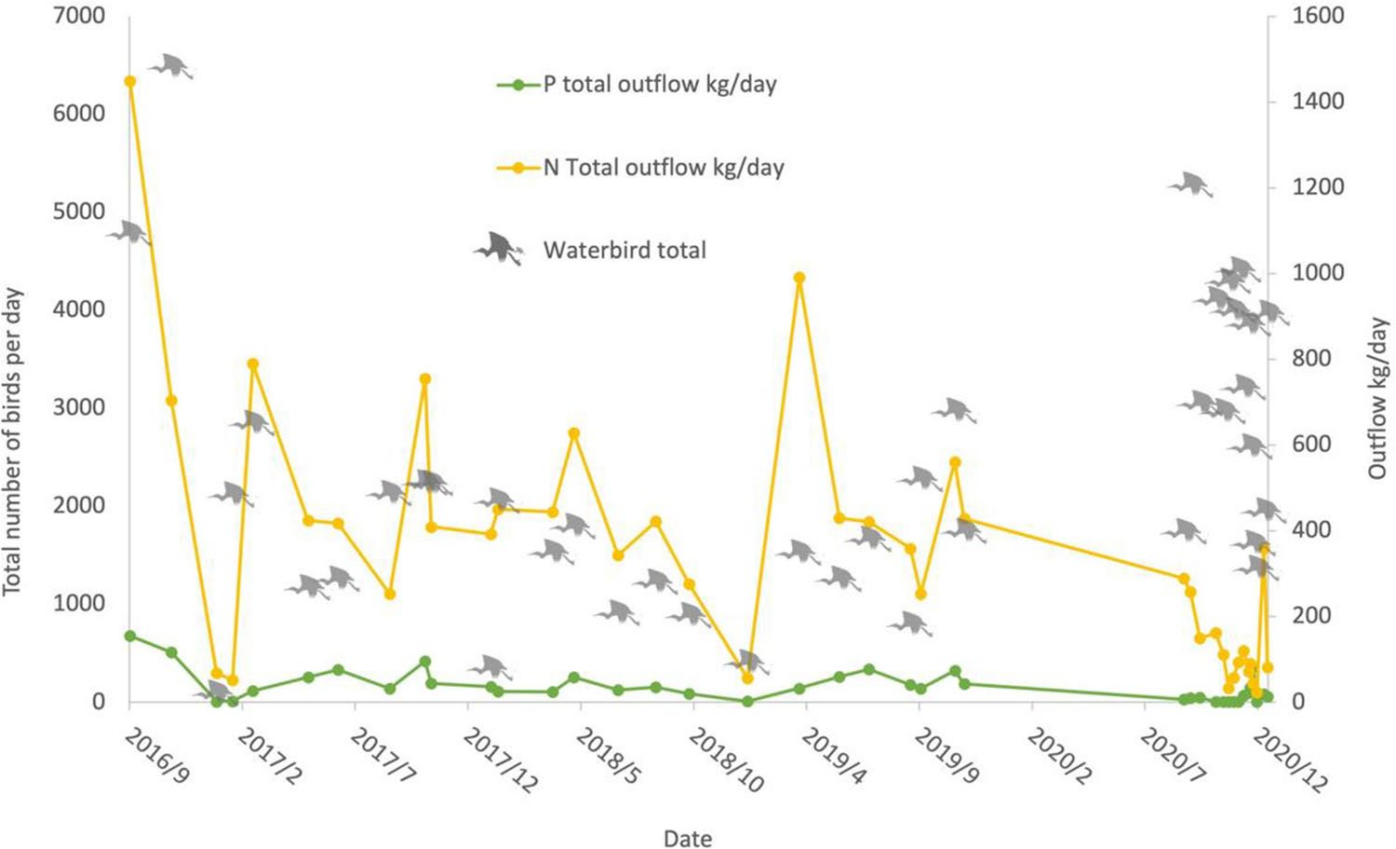
The number of observed birds at Dumbrăvița compared to the rate nutrients (total nitrogen and phosphorus) leaving the Dumbrăvița section of the Homoród stream

**Fig. 2:**
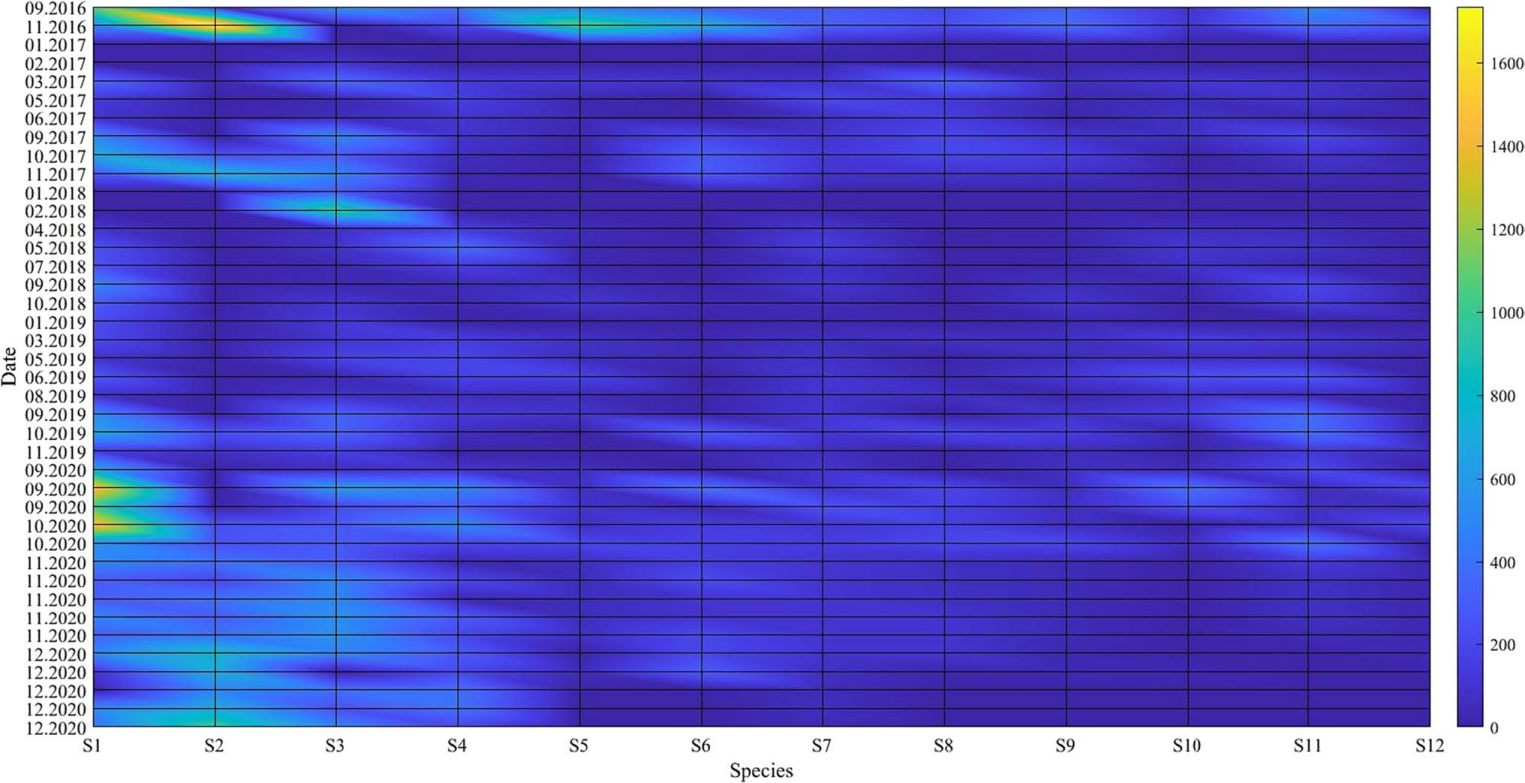
Visual representation of the monthly number of individuals per bird species present at the Dumbrăvița section of the Homoród stream, September 2016–December 2020

Figure 1 displays the correlation between the number of birds present and the nutrient rate within the water. The observed number of birds tends to be higher than average in the autumn–winter migratory period (September–December), while slightly higher than average values can be observed during the spring migratory period (March), less than average is present in July and August, and January has a very low number of birds (Fig. 2).

**Fig. 3:**
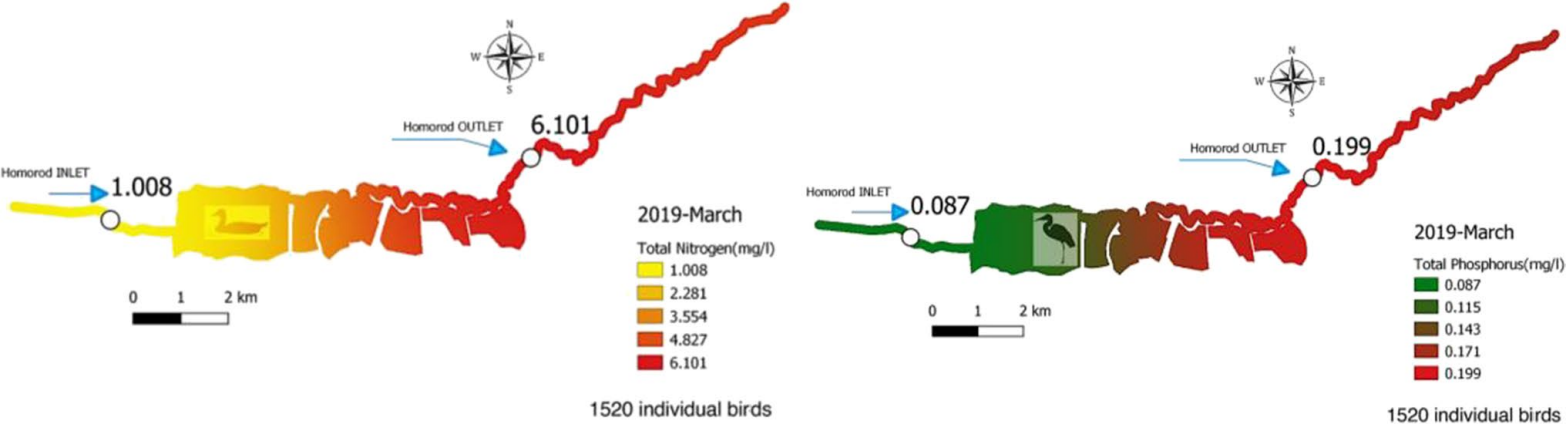
Changes in the level of total nitrogen (mg/l) on the left and the total phosphorus level (mg/l) on the right in March 2019 with 1520 ind. waterbirds the Dumbrăvița section of the Homoród stream

**Fig. 4:**
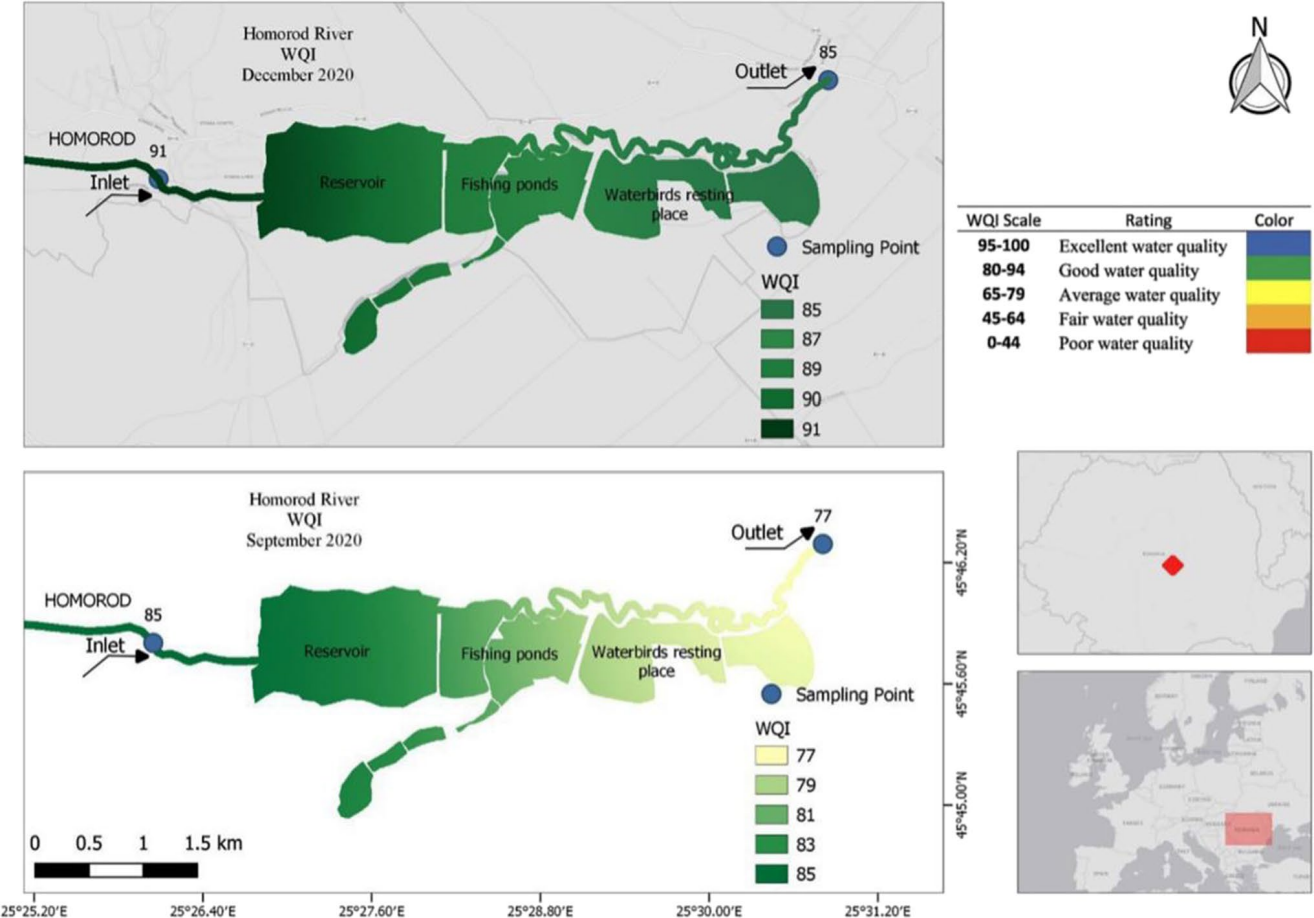
Overview of the study area and spatial distribution and changes in WQI

The figure shows the time series of the observed counts of individuals per bird species vertically and the total number of observed individuals per bird species present at the same time horizontally. The light blue (> 500), green (> 1000), and yellow (> 1500) sections indicate increasing bird abundance. The observed bird numbers coincide with the outlier observations on the total phosphorus and nitrogen outflow, where phosphorus tends to be higher in May and the September–December period, and nitrogen tends to be higher in March and the September–December period. The presence of birds in the area based on their numbers and probable nitrogen and phosphorus contribution is not a determining factor for water quality, but it has a noticeable effect. The changes in the measured parameters are visualized using QGIS and are shown in Figs. 3 and 4.

Figure 3 shows that the area has large changes in individual water parameters between inflow and outflow on the example of a spring migratory period.

Figure 4 shows the observed area and examples of the change in water quality calculated by the WQI technique. The inflowing, always good water quality category water dips lower going through the Dumbrăvița section mainly to average, sometimes even dropping to the fair quality category at the outlet, sometimes remaining in good quality.

**Fig. 5:**
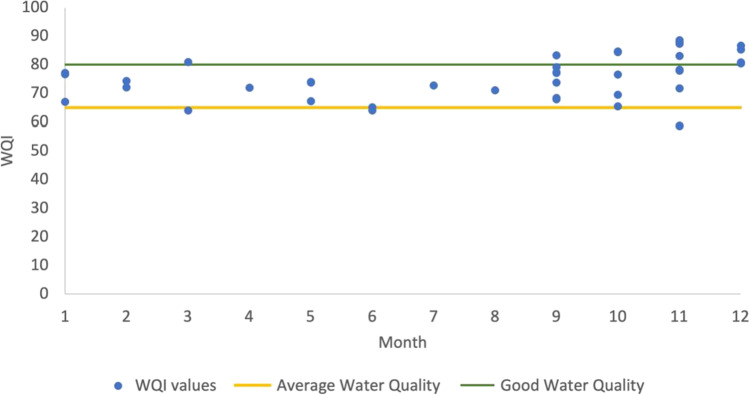
Monthly WQI values in the outflow (Dumbrăvița section of the Homoród stream, 2016–2020)

Figure 5 shows the distribution of monthly WQI values in the outflow from September 2016 to December 2020. As it can be seen, most observed values indicate average or better water quality. However, water quality can deteriorate due to increased nutritional load (both natural and anthropomorphic) as well as environmental changes, such as temperature influencing microbial activity and physicochemical parameters or the changes in flow decreasing the concentration of nutrients. The measured phosphorus and nitrogen concentration, which influenced the WQI values, can be seen in Fig. 5, which reinforce the previously established patterns (elevated values in the spring and the autumn migratory period).

All measurements made by the Olt Water Basin Administration are presented individually according to their official sampling frequency.

Seasonal changes within the chemical parameters of water (Table 1) were evaluated without data on the number of species of migratory birds present to isolate seasons and months with significant outliers in terms of chemical parameters of water. After the eigendecomposition step of PCA, in accordance with Eq. 3. The retained cumulative variance, as well as the scree plot of the transformation, is shown as a function of the PC number in Fig. 12 in the Appendix.

The number of retained PC’s has been chosen as being 2 based on the scree plot’s elbow (Jolliffe 2005). To represent the dataset, the data points have been plotted in the PC subspace based on their calculated coordinates. This is shown in Fig. 13 in the Appendix for the first 2 PCs with the percentage of variance captured for each PC in the axis legend.

**Fig. 6:**
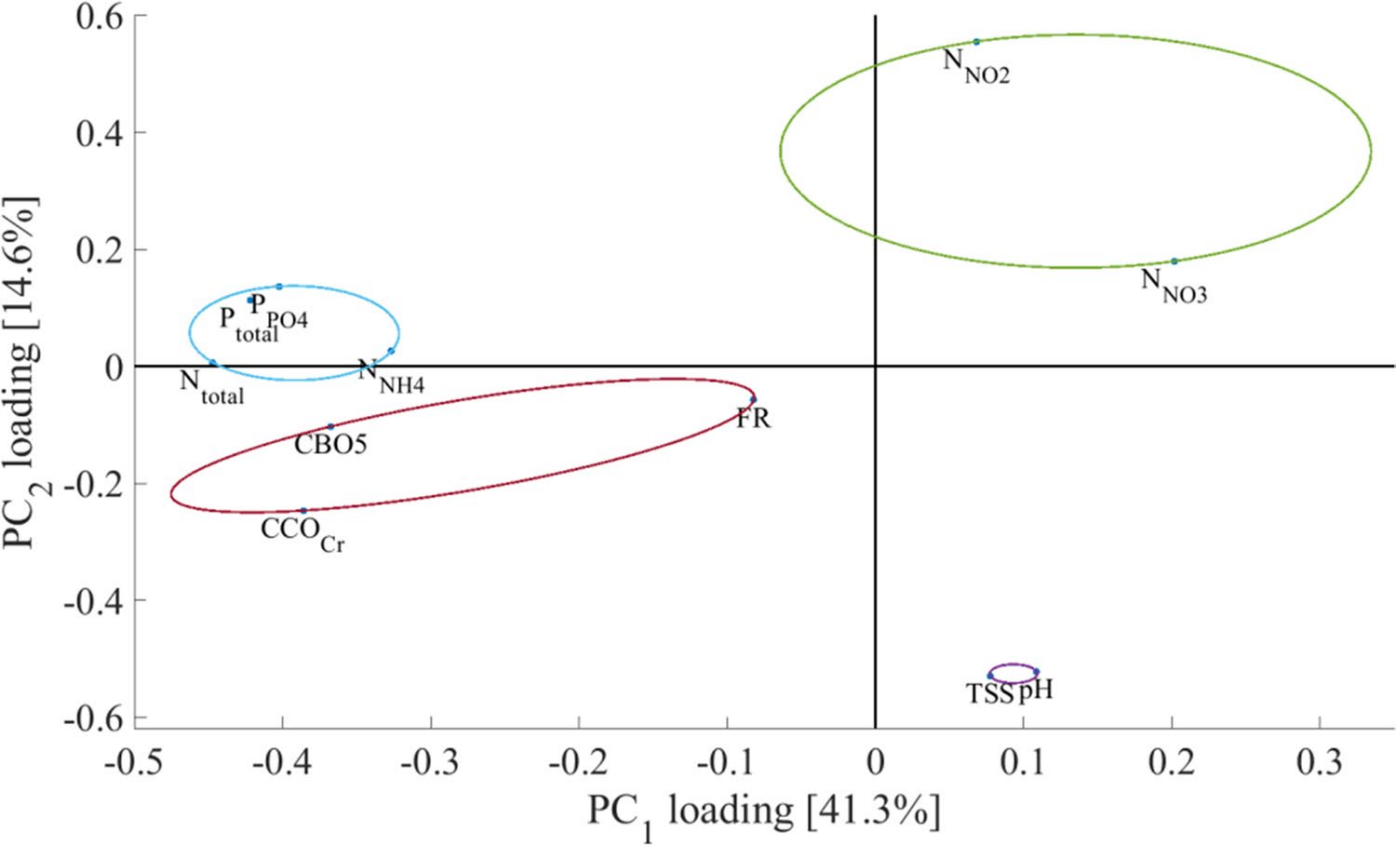
PCA analysis of water parameters (Dumbrăvița section of the Homoród stream, 2016–2020)

The loadings of the PCA transformation (${P}_{\varepsilon }$) are shown in Fig. 6.

The investigated variables have been clustered in the PC subspace through *k*-means clustering based on their loading scores, using squared Euclidean distance. The optimal cluster number was chosen to be four. The four clusters are represented in Fig. 6 as ellipses of different colors. Based on this, the investigated variables shown in Table 1 have been ordered into four groups of variables based on their loading scores.

The first cluster includes the variables of nitrite nitrogen and nitrate nitrogen (N-NO_2_, N-NO_3_) compound concentration within the water. Their similar loading scores within the PC subspace indicate a positive correlation between these variables (Kanownik et al. 2019). Due to their positive loading for both principal components, samples with high nitrite and nitrate compound concentration will have high positive scores in both PC_1_ and PC_2_.

The second cluster, which contains the pH and total suspended solids (TSS) variables, also shows a similar tendency (Akoto et al. 2017), with the difference that samples with high pH and TSS will have great positive PC_1_ and negative PC_2_ scores.

Cluster number three contains the total phosphorous (P_total_) and phosphate (P-PO_4_) concentration within the water, as well as the total nitrogen (N_total_) and ammonium (N-NH_4_) compound concentration within the water. The loading scores of this cluster indicate a straightforward negative correlation between the listed variables and the variables which are members of cluster two. Based on the loading scores of these variables samples with high total phosphorous (P_total_) and phosphate (P-PO_4_) concentration or total nitrogen (N_total_) and ammonium (N-NH_4_), compound concentration tends to have great negative PC_1_ and slightly positive PC_2_ scores. Based on their loadings in PC_1_, the members of cluster number three and cluster number one have a strong negative correlation. This is to be expected since nitrates can reduce sediment phosphorus release through oxidation and by stimulating the growth of phytoplankton (Ma et al. 2021; Padisák and Naselli-Flores 2021).

Cluster four encompasses the variables pertaining to the biological and chemical oxygen demand (CBO_5_, CCO_Cr_) of the water, as well as the concentration of fixed residue (FR). If the dissolved and suspended solids in the water are not oxidable; then, FR will not be closely correlated and can even be reversely correlated to CCO_Cr_ and CBO_5_, while if the contaminants are largely available to microorganisms, all three will be correlated. Based on the loading scores, a straightforward negative correlation can be observed between the variables of cluster number one and cluster number four. In conclusion, it can be expected that samples with high biological and chemical oxygen demand or fixed residue concentration will have great negative PC scores with regard to both PC_1_ and PC_2._

After observing Fig. 13 in the Appendix and the loadings seen in Fig. 6, it can be seen that most of the dataset’s variation stems from unusually large concentrations of phosphorous and phosphate compounds and their correlated variables. These are the data points in Fig. 13 in the Appendix that are far off the negative side of the PC_1_ axis away from the origin.

**Fig. 7:**
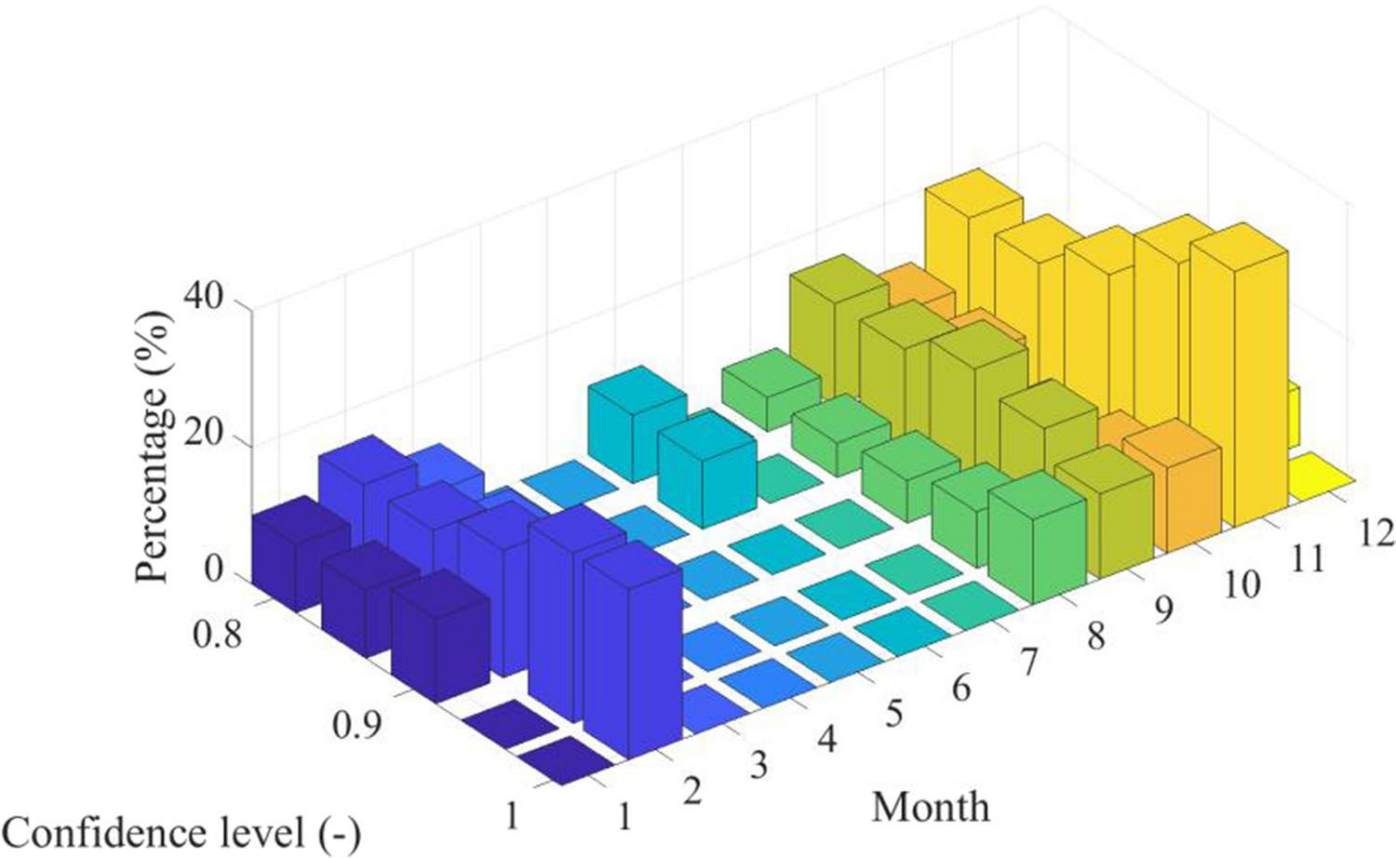
Distribution of the outlier data according to months for various confidence levels (Dumbrăvița section of the Homoród stream, 2016–2020)

To observe which data points are outliers, Hotelling’s *T*^2^ test was conducted according to Eq. 6 and Eq. 7. The time distribution of the identified outliers was studied. The distribution of outliers among the months of the year for various confidence levels is displayed within Fig. 7.

Based on the results of Fig. 7, most of the outliers for all confidence levels lie in the months of January (on average 17% of outliers) and November (on average 35% of outliers). It can be noted that the outliers are all mostly distributed among months where there is either prominent migration of birds (September–November as well as January–March) or time periods where due to the melting of snow caps, large quantities of water enter the lake (January–March).

Based on the results, it was concluded that the migration of birds strongly correlates with the annual fluctuation of phosphorous and nitrogen compounds and should be taken into account as a factor for establishing bounds for water quality metrics.

### Correlation between the presence of migratory bird species and water quality

The list of investigated species and their later reference codes are shown in Table 2. The table also shows the estimated nitrogen and phosphorous compound concentration within the excrement of each individual species based on literary references (Hanson, et al. 2006).

**Table 2:** Daily nitrogen and phosphorous loading of bird excrement by species

Code	Species	Scientific name	Nitrogen load (g day^−1^ individual^−1^)	Phosphorous load (g day^−1^ individual^−1^)	Source
S1	Black-headed gull	*Chroicocephalus ridibundus*	0.88	0.96	(Gwiazda 1996)
S2	White-fronted goose	*Anser albifrons*	0.69	0.08	(Kear 1963)
S3	Mallard	*Anas platyrhynchos*	2.62	0.42	(Gwiazda 1996)
S4	Eurasian coot	*Fulica atra*	0.61	0.19	(Boros et al. 2008)
S5	Great cormorant	*Phalacrocorax carbo*	1.04	4.58	(Marion et al. 1994)
S6	Eurasian teal	*Anas crecca*	0.58	0.18	(Boros et al. 2008)
S7	Gray heron	*Ardea cinerea*	1.38	3.78	(Marion et al. 1994)
S8	Pochard	*Aythya ferina*	0.61	0.19	(Oláh 2003)
S9	Great egret	*Casmerodius albus*	1.38	3.78	(Marion et al. 1994)
S10	Great crested grebe	*Podiceps cristatus*	0.61	0.19	(Oláh 2003)
S11	Caspian gull	*Larus cachinnans*	0.66	0.62	(Gould and Fletcher 1978)
S12	Shoveler	*Anas clypeata*	0.58	0.18	(Oláh 2003)

Similarly to the previous investigations, PCA was applied to the historical dataset, which contained information on the number and species of birds observed during different months of the year as well as the previously explored indices of the chemical parameters of water shown in Table 1. This meant the observation of the historical data as a function of 23 variables. The number of PCs was chosen as per in the previous subsection based on the results of Fig. 14 in the Appendix. In this case, the data has been compressed into 5 PCs.

**Fig. 8:**
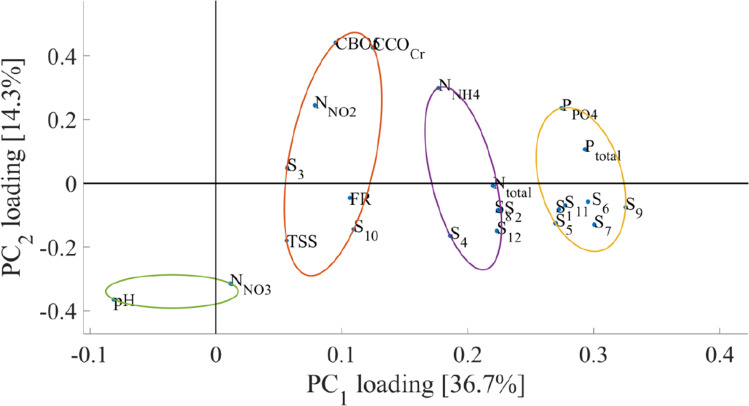
PCA analysis of water parameters and bird data (Dumbrăvița section of the Homoród stream, 2016–2020)

The samples in the 2D PC subspace are shown in Fig. 15 in the Appendix. The loadings of the PCA transformation in the 2D PC subspace are shown in Fig. 8.

Based on the loadings of Fig. 8, the correlation between the chemical parameters of water and the number of individuals per species was explored. Clustering was performed similarly to the previous subsection. The optimal number of clusters was determined to be four which are shown with differing colors.

Within the observed Dumbrăvița region and period the black-headed gull, great cormorant, eurasian teal, gray heron, great egret, and caspian gull (*Chroicocephalus ridibundus*, *Phalacrocorax carbo*, *Anas crecca*, *Ardea cinerea*, *Casmerodius albus*, and *Larus cachinnans*) show similar PC_1_ loadings as the variables pertaining to total phosphorous as well as phosphate compound concentration within the water (Marion et al. 1994). The eurasian teal *Anas crecca* has benthivorous feeding habits and produces excrements with a low quantity of phosphorus; however, they tend to dabble about on mudflats or places covered with two or four centimeters of water (Olney 1962), allowing phosphorus to be released from the sediment into the watercourse (Hanson et al. 2006). These variables make up cluster number one. It is safe to assume that there is a straightforward positive correlation between the number of individuals per said bird species and the chemical parameters of water, which could point to a significant environmental impact. The similar loading scores between these bird species also indicate a correlation between them as well, pointing to similar migration and habitation patterns. This can be explained by excess phosphorus in the feces of the listed waterbird species. This is especially noticeable between the black-headed gull and caspian gull.

The second cluster includes white-fronted goose, coot, pochard species, and shoveler (*Anser albifrons*, *Fulica atra*, *Aythya ferina*, and *Anas clypeata*), which have similar loadings with regard to PC_1_ as the first group, albeit with smaller absolute values. Based on the PC scores, a straightforward correlation between the presence of the birds within this cluster and the total nitrogen compound concentration can be observed. The excrement of herbivorous waterfowl contains more nitrogen (Boros 2021).

The first cluster includes the mallard and the great crested grebe species (*Anas platyrynchos* and *Podiceps cristatus*). The variables describing the individual number of the species have similar loadings in regards to PC_1_ and PC_2_ that differentiate them from the previous groups. Based on the clustering results, the mallard (*Anas platyrhynchos*) and great crested grebe (*Podiceps cristatus*) species are correlated to the total suspended solids, fixed residue, biological oxygen demand, and nitrite compound concentration within the water. This is explained by the high number of individuals and feeding habits (Gwiazda et al. 2014). Gwiazda and colleagues provides a reference for the mallard (*Anas platyrhynchos*) species foraging for food, disturbing the sediment and they are present in very large numbers, which feasibly would provide a large part of the explanation for this group. The great crested grebe (*Podiceps cristatus*) is present in much smaller numbers, its feeding habits are different, and they are diving for their prey; however, (Piersma et al. 1988) writes that for shallow lakes, depending on the composition of the fish population, a large portion of the grebes’ food comes from the bottom layer, so it is feasible that they also contribute some to the resuspension of the nutrient rich bottom sediment while chasing fish, even though they are not foraging in the mud.

The fourth cluster includes the variables describing the pH of the water as well as the concentration of nitrate nitrogen (N-NO_3_) compounds. Since no bird species were included in this cluster, this indicates that the presence of the investigated migratory birds had little to no effect on these variables. Since bird feces is thought to contain magnesium ammonium phosphate and ammonium urate or, in the case of some earlier sources, uric acid or uric salts, the nitrate-nitrogen concentration in the water will be subject to the residence time of the excreted nitrogen and the nitrification process, explaining why it is not in the same group as the bird species (Crouch et al. 2020). Higher nitrate concentrations may lower the pH of water. Thus, the two can be associated, but the pH can also be changed by other factors, such as dissolved carbon dioxide or ammonia (Glass and Silverstein 1998).

**Fig. 9:**
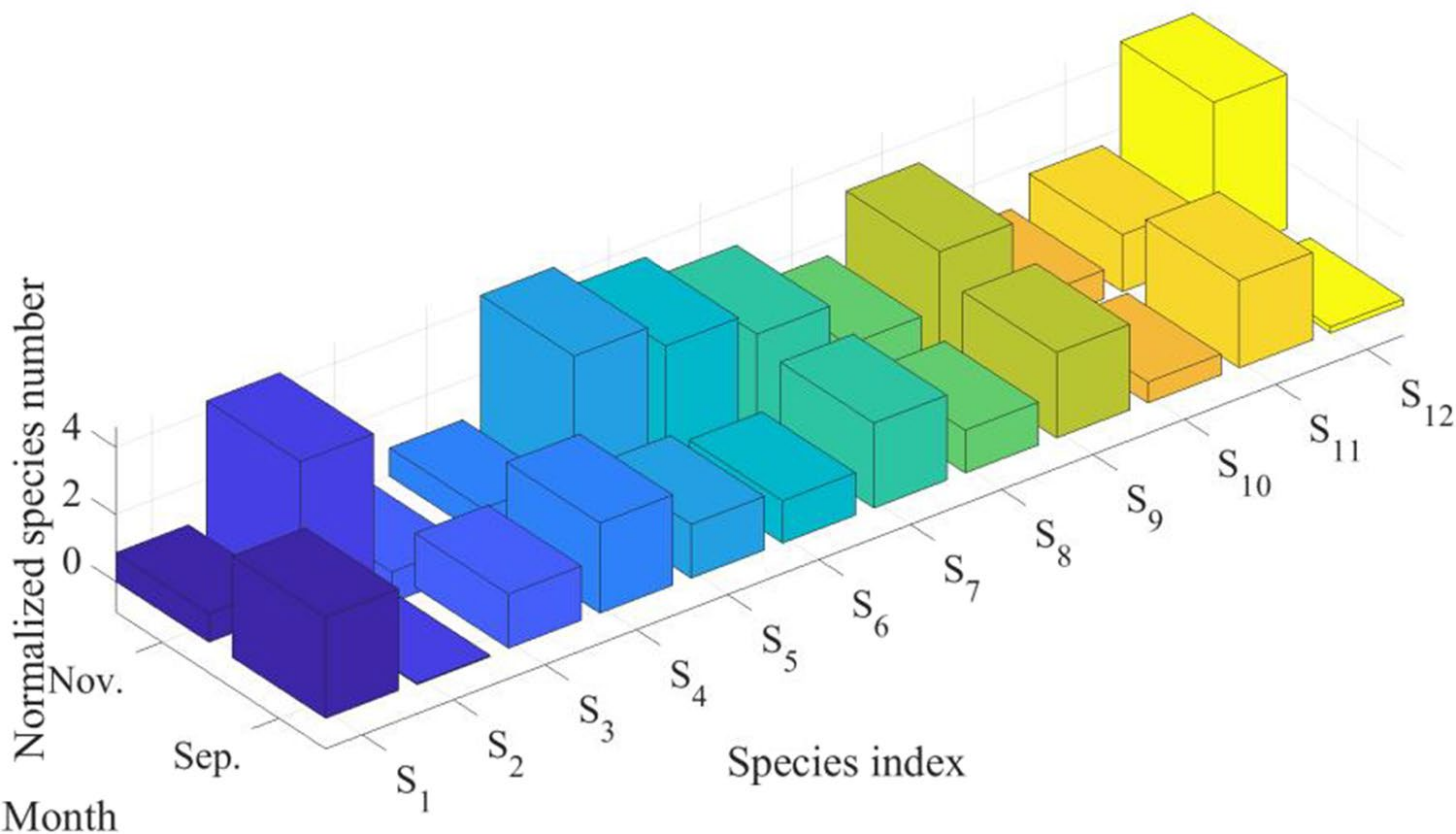
Standardized bird species number within the outlier samples

**Fig. 10:**
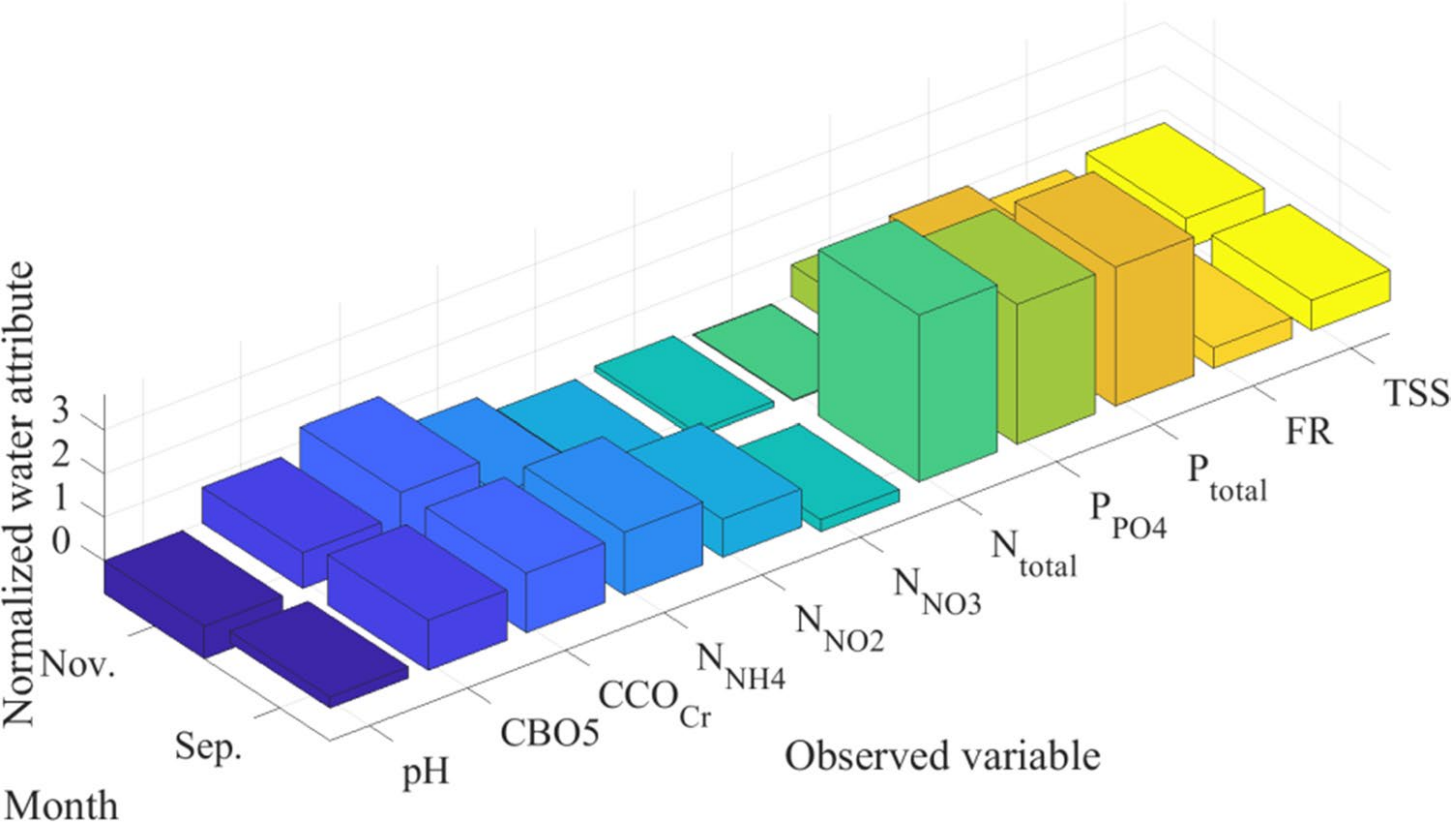
Standardized chemical parameters of water within the outlier samples

In order to identify outliers within the dataset, Hotelling’s *T*^2^ test has been utilized with a 95% confidence interval. After evaluation, two samples have been found to be outliers (data points with PC coordinates (8.35, -1.77) and (8.23, 0.84) in Fig. 15 in the Appendix, respectively). Data about these outliers have been collected and displayed in Figs. 9 and 10.

After observing the figures, it can be seen that the outliers are within the months of November and September, months which account for the majority of outliers with regards to variance within chemical parameters of water (Fig. 7).

The first outlier can be seen in September, which has PC coordinates of (8.23, 0.84) in Fig. 15 in the Appendix. High positive values within PC_1_ and PC_2_ point to the presence of an above-average number of the species within cluster one and two and possibly high concentrations of total phosphorous, nitrogen, and phosphate compounds based on the loading scores seen in Fig. 8. This can be verified after observing Figs. 9 and 10. Figure 9 shows that the normalized value of the number of species of cluster one and cluster two was quite above average in September, and Fig. 10 verifies that this caused above-average concentrations of total nitrogen, phosphorous, and phosphate compounds.

In the outlier seen in November, it can be observed in Fig. 9 that there was a great increase in the observed number of the white-fronted geeses, great cormorant, eurasian teal, gray heron, great egret species, and shoveler. This outlier point is represented by coordinates (8.35, -1.77) in Fig. 15 in the Appendix. Referring to the loadings in Fig. 8, all of the listed bird species have positive loadings for PC_1_ and negative loadings for PC_2,_ which explains the PC score. After observing the normalized chemical parameters of water in Fig. 10, it can be seen that all variables except pH, nitrite, nitrite nitrogen, nitrate nitrogen, ammonia nitrogen, and total nitrogen concentrations are slightly above 0, indicating above-average values due to the loading within the bird excrement. The presence of birds does not completely explain the nitrogen load in the water. The outflow has a higher nitrogen concentration than would be expected from bird droppings and the flow rate.

After observing the normalized chemical parameters of water in Fig. 10, it can be seen that all variables except pH, nitrite nitrogen, nitrate nitrogen, ammonia nitrogen, and total nitrogen compound concentrations are elevated, indicating above-average values. This corresponds to the load within the bird excrement because the number of species and individuals associated with phosphorus also markedly increases in this time period due to migration. However it must be noted that since standardized values were used data about the absolute number of birds within each month is not explicitly seen. The total number of birds in September was much greater than the number of birds observed within November. This leads to heavy increase of specific P and N compounds within the water.

**Fig. 11:**
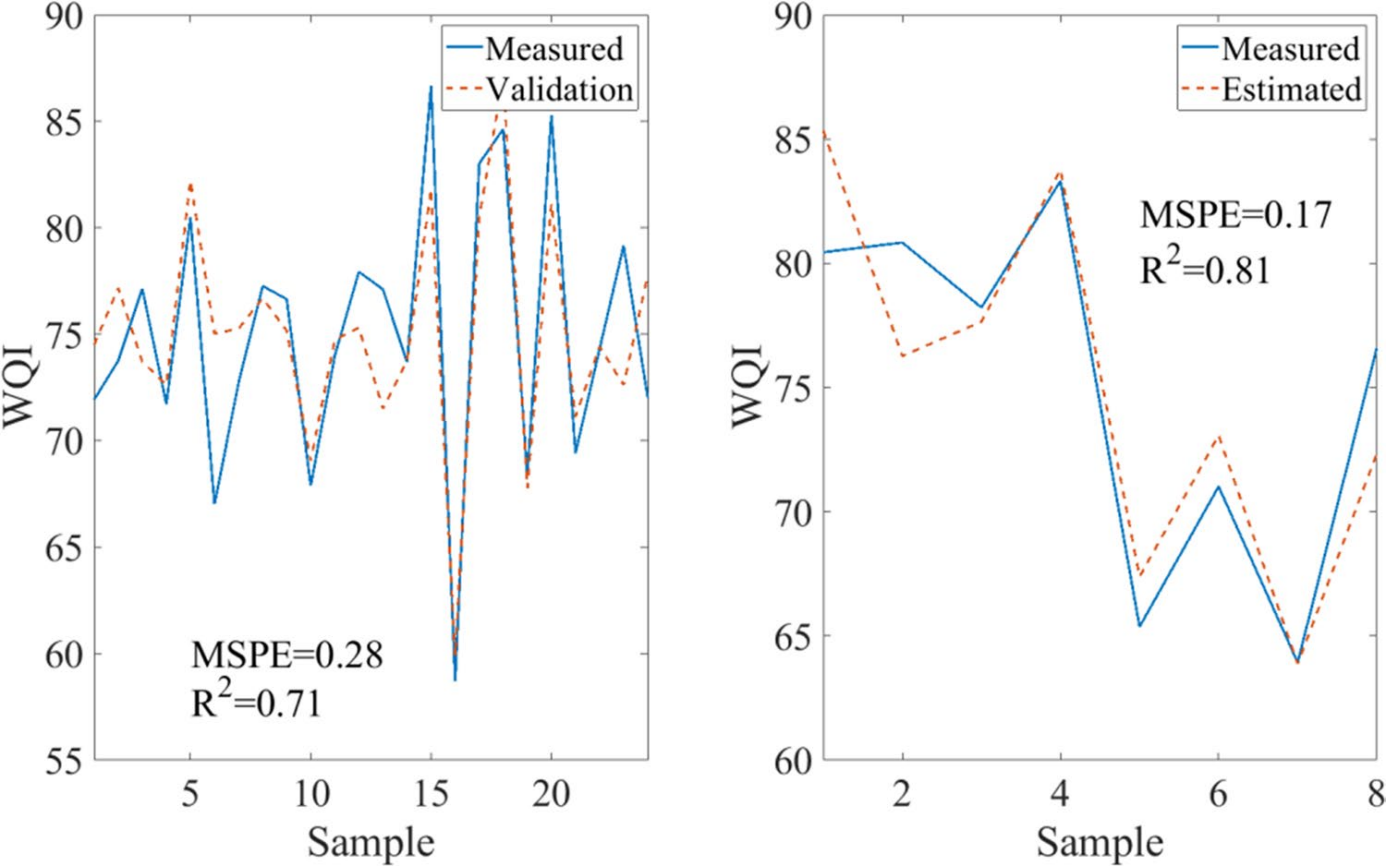
(**a**) Training data set for estimation of WQI based on bird number. (**b**) Validation data set for the prediction of WQI based on bird number

**Fig. 12:**
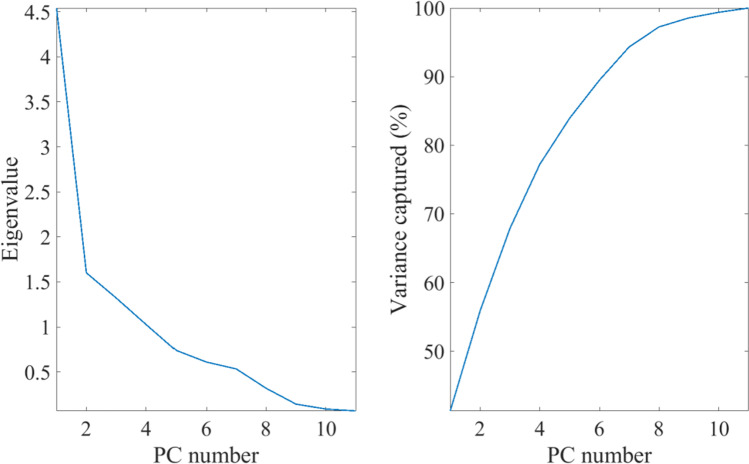
(**a**) The scree plot of the PCA transformation. (**b**) The retained variance within the principle components

**Fig. 13:**
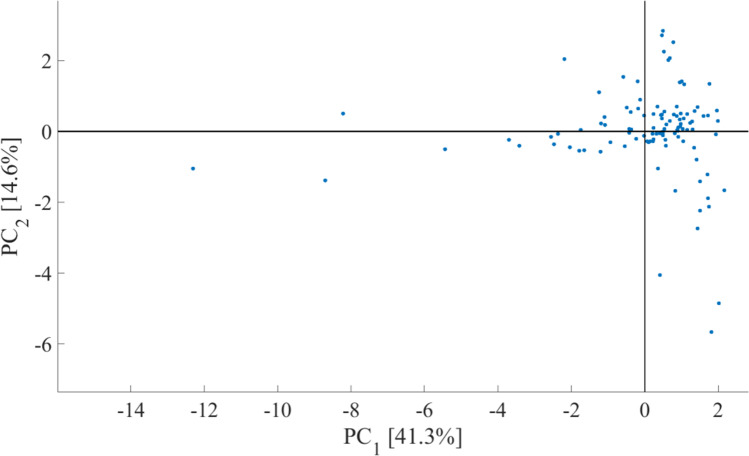
Location of the observed data points within the PC subspace

**Fig. 14:**
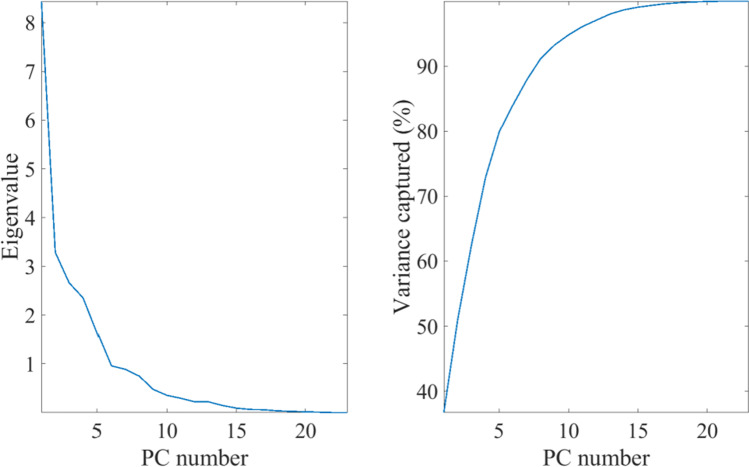
(**a**) The scree plot of the PCA transformation. (**b**) The retained variance within the principle components

**Fig. 15:**
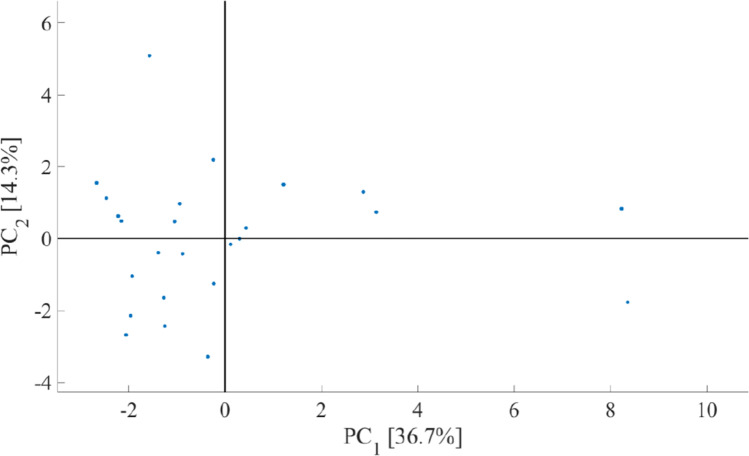
Data points within the 2D PC subspace for the enhanced dataset

To provide a link between the presence of the 12 investigated bird species and the WQI value of the water PCR was employed. For the regression, the variables displayed in Table 1 were utilized. The observed variable was the WQI value of the water for each investigated time sample. A regression was identified for the data set and used for the prediction of changing WQI with regard to the presence of migratory birds. Both the training and validation data set, as well as the fit results, are shown in Fig. 11. Training and validation data sets were obtained by randomly splitting the original observed data set in a 25–75% ratio. 25% of the data set was used as validation data, while 75% was used as training data.

Based on the results of Fig. 11, PCR managed to establish an accurate estimation of the WQI using the number and species of the birds present with relatively high *R*^2^ and low MSPE scores. In the case of the training data, the *R*^2^ score was 0.71, while the MSPE was 0.28. For the validation data set, the metrics were 0.81 and 0.17, respectively. The consistency of both measures and their favorable values indicate robust and good performance of the PCR model for WQI estimation.

## Conclusion

The PCA method was utilized for comparing various waterbird species with different numbers of individuals, water quality stream parameters, and the temporal relationship of nitrogen and phosphorus loads.

Our model demonstrated the significant role of piscivorous waterbirds in nutrient loading, specifically phosphorous content. This could be validated since the diet and feces of carnivorous waterbirds contain a relatively high phosphorous concentration. Their joint phosphorous contribution may significantly modify the often phosphorous-limited wetland nutrient loads. On a regional scale, the effects may even be more significant and not only with respect to phosphorous but also nitrogen (Karl 2000).

When incorporating the seasonal abundance of waterbirds, our model suggests that carnivorous waterbirds, such as the great cormorant, are only of minor importance for nitrogen loading into the Dumbrăvița watercourse, while the nitrogen contribution of a large number of herbivorous geese is more significant, but the phosphorus load is also significant because it is coupled with the feeding habits of duck species, such as eurasian teal (*Anas crecca*), which forage for mollusks in shallow water (Domşa et al. 2014). The eurasian teal (*Anas crecca*) and mallard (*Anas platyrhynchos*) species are present in very large numbers at the same time, foraging for food, disturbing the sediment, which feasibly would provide part of the explanation for this group by the resuspension of the nutrient rich bottom sediment.

Based on the results of PCA, a regression was performed to provide a method for means of predicting changes within WQI as a function of the presence of migratory birds. The estimated model managed to predict the changes within WQI accurately with an *R*^2^ value of 0.81 and MSPE of 0.17.

The results can contribute to the water management plans of different water bodies and may be incorporated into the expected consequences of bird conservation measures, as significant changes in the number of individuals can greatly affect the phosphorus and nitrogen concentrations in a given water body. The influence of the joint phosphorous contribution of piscivorous and herbivorous water birds may be significant (up to 6.6% of P at the outflow) on the often phosphorous-limited wetland nutrient loads, while the nitrogen contribution dominated by herbivorous geese is limited (up to 0.7% of N at the outflow) in the case of Dumbrăvița. The relationships identified can be used to prepare a decision-support algorithm that predicts the impact of expected changes in bird numbers from bird conservation measures for the water management plan, so that it can be prepared to take into account the magnitude and periodicity of the changing nutrient load.

As a line of further research, the use of non-linear multivariate statistical methods such as kernel PCA, non-linear PCA, non-linear PLS, or neural networks may be utilized to more accurately model and predict changes within water quality metrics as a function of migratory bird presence. Additionally, the use of computational fluid dynamics methods may be combined with the previously mentioned methods to identify the spatial distribution of pollution agents within water bodies and to predict the impact of changes to the water body on the water quality.

## Data Availability

Data not included in the manuscript will be made available on request. Restrictions apply to the availability of the raw water quality parameters measured by the Olt Water Basin Administration (Administrația Bazinală de Apă Olt) during the period 2010–2019, which can be made available only after approval by the Inspectorate.

## Appendix

Figures 12, 13, 14, and 15
